# Immediate effects of Thai foot massage on renal blood flow, psychological stress, and heart rate variability in community-dwelling older adults: a randomized controlled trial

**DOI:** 10.12688/f1000research.148453.1

**Published:** 2024-07-26

**Authors:** Yada Thadanatthaphak, Jaturat Kanpittaya, Wittawat Takong, Sutin Chanaboon, Kukiat Tudpor

**Affiliations:** 1 Department of Health and Sport Science, Mahasarakham University, Maha Sarakham, Thailand; 2 Department of Radiology, Faculty of Medicine, Khon Kaen University, Khon Kaen, Thailand; 3 Neuroscience Center, Bangkok Pattaya Hospital, Chonburi, Thailand; 4 Sirindhorn College of Public Health, Khon Kaen, Thailand; 5 Faculty of Public Health, Mahasarakham University, Maha Sarakham, Thailand

**Keywords:** Foot massage, renal blood flow, parasympathetic activity, heart rate variability, autonomic nervous system.

## Abstract

**Background:**

Renal blood flow (RBF) is regulated by an autonomic nervous system and is reduced in older adults. Massage has been previously found to increase blood flow.

**Objective:**

This two-armed double-blind, randomized controlled trial aimed to investigate the immediate effects of Thai foot massage (TFM) on RBF, psychological stress, and heart rate variability (HRV) in older adult persons.

**Material and Methods:**

The 26 healthy older adult volunteers were recruited and randomly assigned to the TFM group (13 persons) and the control group (13 persons). The TFM group received a 15-minute Thai foot massage, and the control group received a 15-minute bed rest. Primary outcomes – RBF parameters [peak systolic velocity (PSV), end-diastolic velocity (EDV), resistive index (RI), volumetric arterial blood flow (VF)] and secondary outcomes – HRV parameters [standard deviation of the normal-to-normal intervals (SDNN), root mean square of successive differences (RMSSD), high frequency (HF), low frequency (LF), and low frequency per high frequency (LF/HF)] were measured after each intervention.

**Results:**

Results showed that the VF significantly increased after TFM (P < 0.05) but not in control. Meanwhile, the stress index significantly reduced after TFM (P < 0.05). SDNN and RMSSD, the proxies of parasympathetic activity, also significantly increased in the TFM group (p < 0.05). Only RMSSD was significantly enhanced in the control group. No side effects were observed.

**Conclusion:**

The TFM could increase RBF and alleviate psychological stress through parasympathetic activity actuation. Therefore, this intervention might improve RBF and relieve stress in the older population. Further study should be carried out on a larger population.

## Introduction

Renal blood flow (RBF) plays a crucial role in maintaining an oxygen supply capable of meeting the demands of renal function and driving glomerular filtration by supplying sufficient capillary pressure. ^1^ Aging affects every tissue and organ in the human body, including the kidneys. The main features of the structural and functional changes that come with kidney aging are a shrinking in size, a decline in the number of functioning glomeruli, and abnormalities in the blood vessels. ^2^ It was discovered that the RBF decreased by around 10% every decade beyond the age of 40, with an average reduction of approximately -85 mL/min each decade across various investigations. ^1^

Factors influencing the RBF include circadian cycle, food intake, smoking, alcohol consumption, medications, physical exercise, and mental stress. According to a reference work entry by Solomon, mental stress or psychological stress is defined as “a form of stress that occurs because of how events in one’s external or internal environment are perceived, resulting in the psychological experience of distress and anxiety ^3^”. Psychological stress can be determined by heart rate variability (HRV), which provides readouts of both sympathetic and parasympathetic variables as well as the stress index. ^4^ It has been evidenced that psychological stress reduces the RBP through sympathoadrenal excitation-mediated vasoconstriction. ^1^^,^^5^^,^^6^ Thus, the factors altering psychological stress are implied to modify the RBF.

Thai massage is a distinct form of therapy that involves deep tissue massage with the thumbs, frequently paired with post-session muscular stretching. Benefits of the massage include improved skin warmth and blood flow, decreased anxiety, decreased depression, decreased sympathetic activity, increased parasympathetic activity, and reduced cortisol and salivary α-amylase levels, indicators of psychological stress. ^7^ The Thai foot massage (TFM) has recently increased RBF in young people. ^8^ Therefore, this study aimed to investigate the immediate effects of Thai foot massage on RBF and HRV in older adults.

## Methods

### Study design and participants

**Figure 1.**
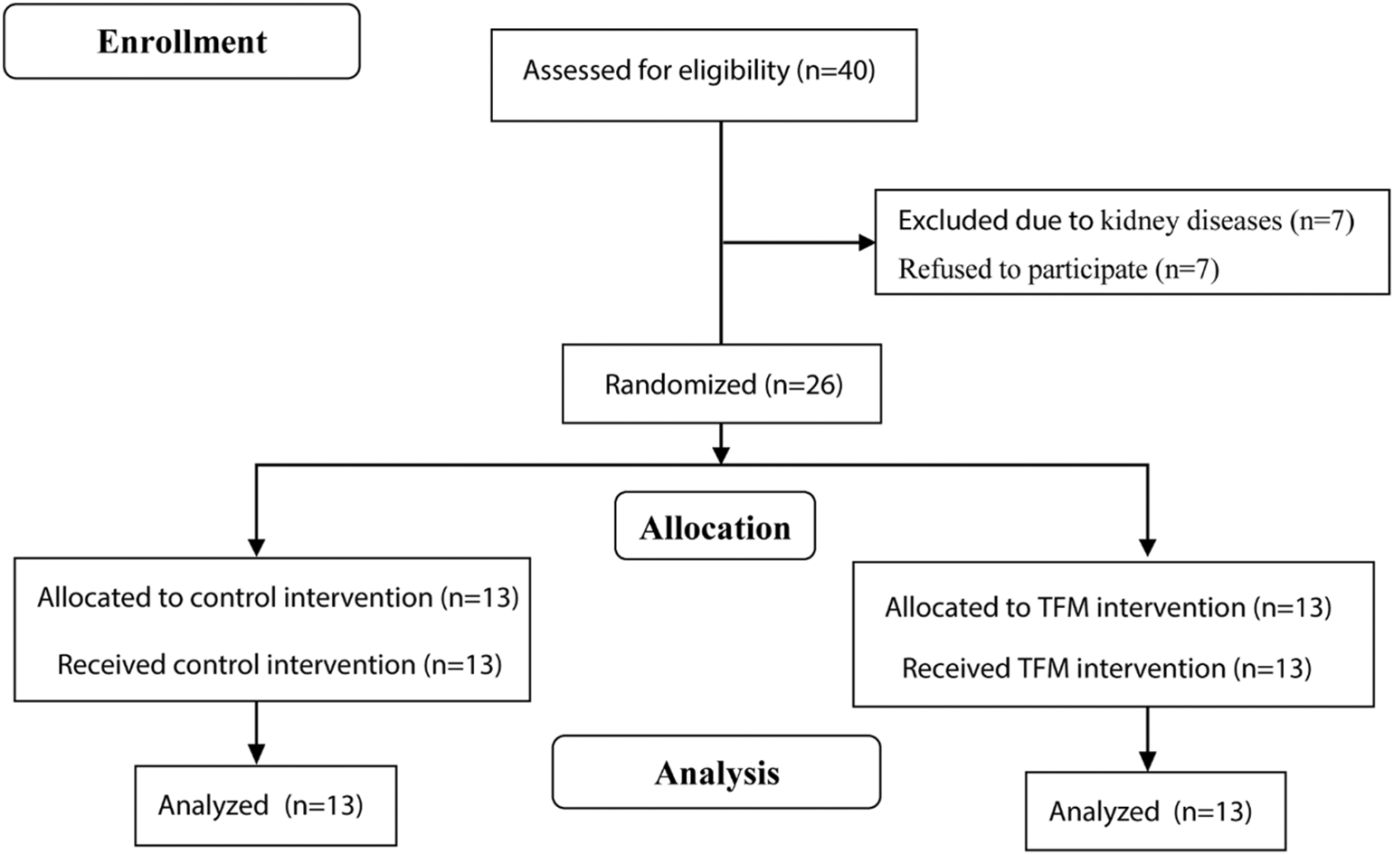
CONSORT diagram depicting experimental design and flow of participants through the RCT.

This study was a two-armed double-blind, randomized controlled trial (RCT). Physical examinations, including blood pressure and urine tests, were performed at Suddhavej Hospital, Faculty of Medicine, Mahasarakham University, to measure the basal health status of the volunteers. The experiments and the measurement of renal blood flow and HRV were conducted in the ultrasound room, where temperature and humidity were set at 25 °C and 60-70%, respectively, at Srinagarind Hospital, Khon Kaen University, a tertiary care university medical center. Written informed consent forms were obtained from all patients. The eligibility criteria for participants were age 60 and older and healthy (not diagnosed by physicians with chronic diseases). Forty older adult volunteers were recruited. Seven volunteers were excluded due to kidney stones, while the other seven refused to participate in the study for personal reasons. The study was carried out between June and December 2017. Repositories of the data can be found at https://osf.io/yrf3j/. ^9^ The research report complies with the Consolidated Standards of Reporting Trials (CONSORT) ( Figure 1). A copy of the original trial protocol, a completed CONSORT checklist, and a flow diagram can be found in the Extended data. ^9^ The study has been approved by the Thai Clinical Trials Registry (TCTR), which became the primary registry of WHO on August 7, 2013 (https://www.thaiclinicaltrials.org/). Our TCTR identification number is TCTR20240526002 ( https://www.thaiclinicaltrials.org/show/TCTR20240526002). There were no changes to methods after trial commencement.

### Ethical approval

All procedures have been registered to the Thai Clinical Trials Registry (TCTR20240526002), approved by the Institutional Review Board (Name: Ethical Review Committee for Human Research, Mahasarakham University, Thailand) with the approval number 014/2559, 29 ^th^ May 2017 and carried out following the ethical principles in the Declaration of Helsinki. All participants signed a consent form stating that data are blinded and exclusively available for professional research staff.

### Interventions

**Figure 2.**
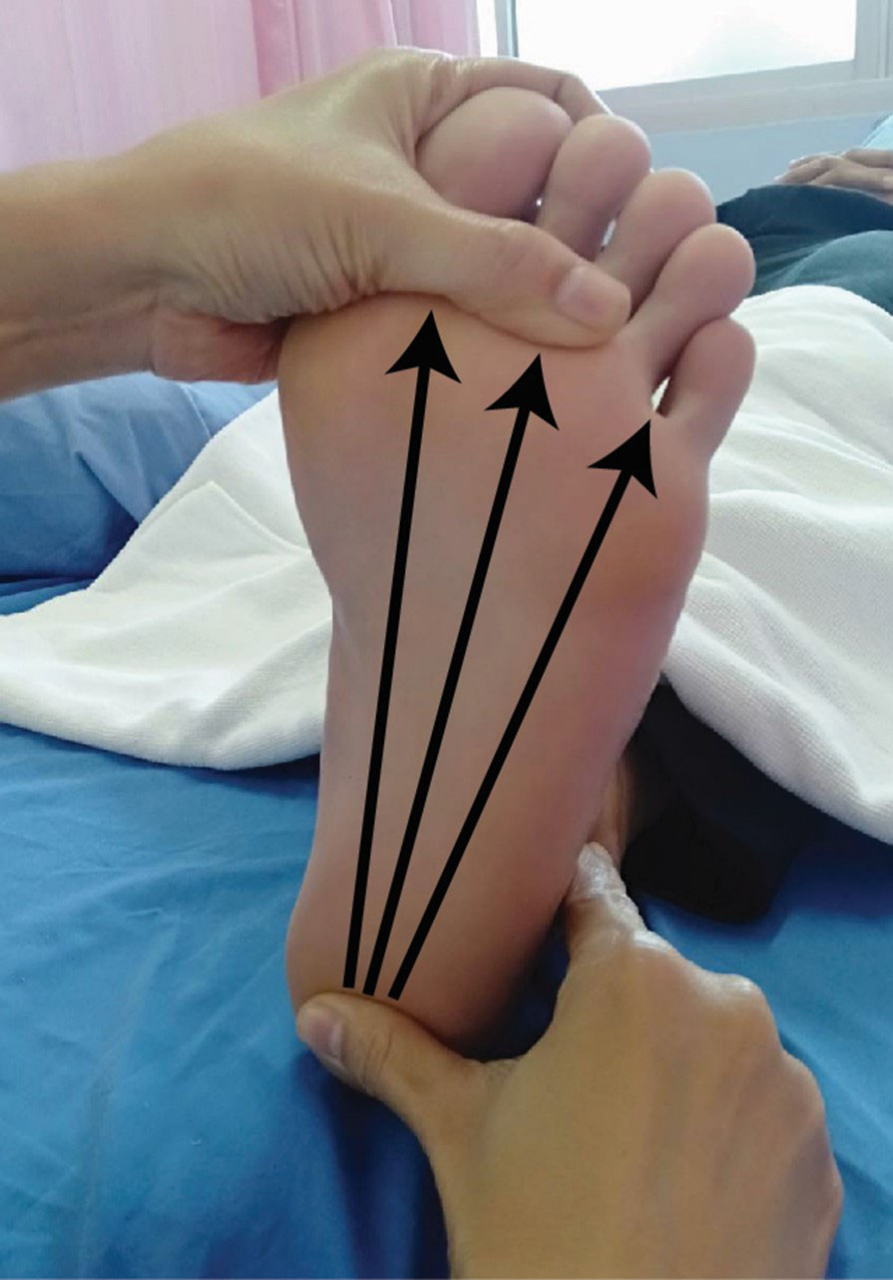
The lines of Thai foot massage.

Before the intervention, the participants were instructed to acclimate themselves by spending 15 minutes in the supine lying posture in bed. Renal hemodynamics (RBF) were measured for 2.5 minutes after the ultra-short-term HRV values were collected. Each participant underwent a blinded, simple random sampling by drawing the assigned number from an indistinguishable container. Y.T. generated the random allocation sequence, while K.T. enrolled and assigned participants to interventions. The experimental group received a 15-minute Thai foot massage using moderate thumb press along three lines on both feet in a supine lying position as previously described ^10^ ( Figure 2). The control group was mandated to rest for 15 minutes. The HRV measurements and RBF were reevaluated right away following the intervention by J.K. and W. T, blinded from the group assignment.

### Outcomes

**Figure 3.**
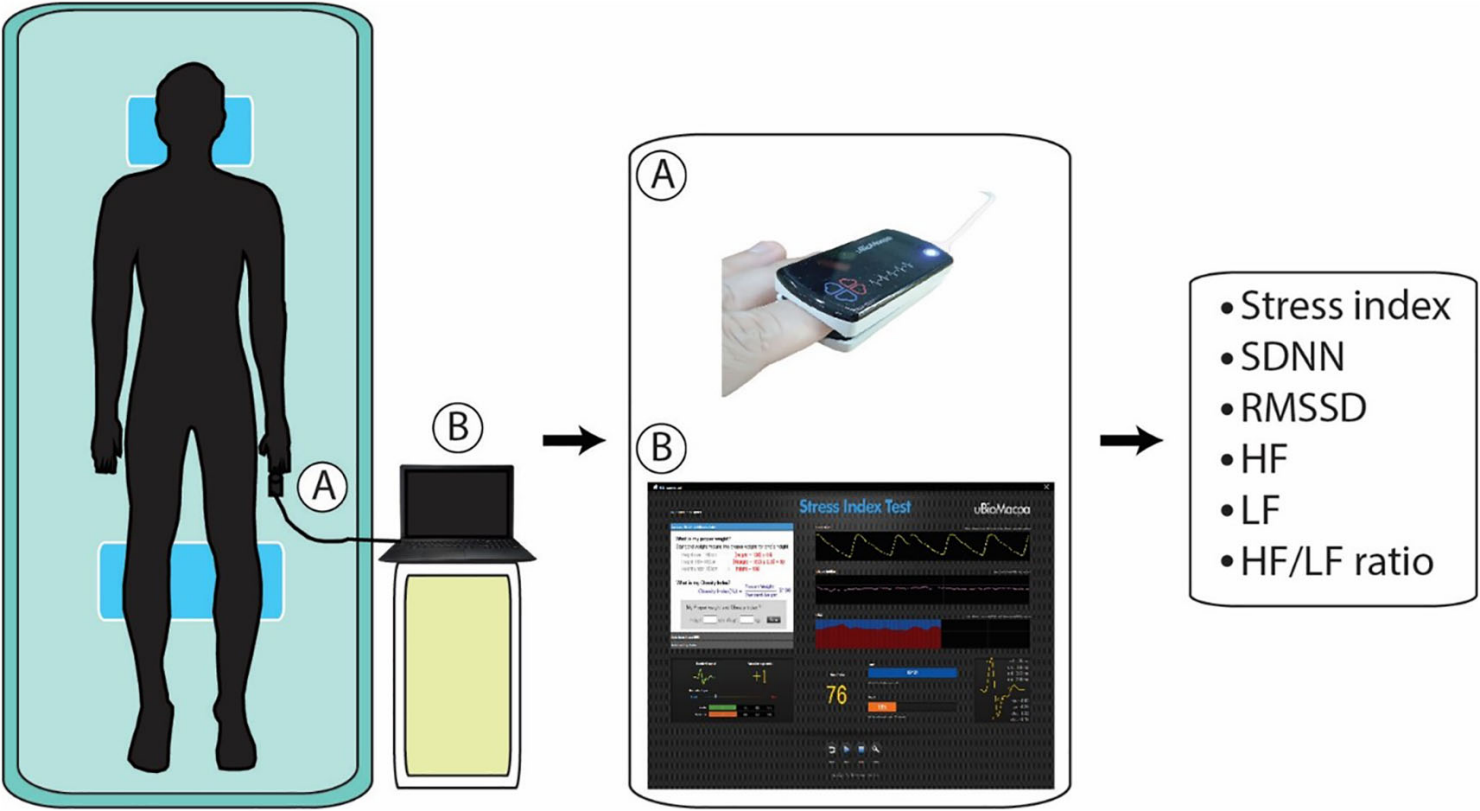
Stress index and heart rate variability measurement. A, photoplethysmography; B, uBioMacpa software.

As previously mentioned, ^11^ photoplethysmography (PPG) on the participant’s left index fingertips coupled to the computer was used to measure the stress index and HRV parameters for 2.5 minutes by a physical therapist using uBioMacpa software version 1.0 (BioSense Creative Co., Ltd, Korea) ( http://www.ubionet.com) ( Figure 3). This software is proprietary and a free alternative is the Kubios HRV Scientific Lite. The stress index scores were divided into five groups: chronic stress (≥ 60), accumulative stress (45–59), primary stress (35–44), temporary stress (25–34), and no stress (< 25). The parasympathetic proxies uBioMacpa (high frequency (lnHF), a standard deviation of all normal R-R intervals (SDNN), and the square root of the mean of the squared successive differences in R-R intervals (RMSSD) are among the sympathetic proxies (low frequency (lnLF) and low/high-frequency ratio (LF/HF ratio).

**Figure 4.**
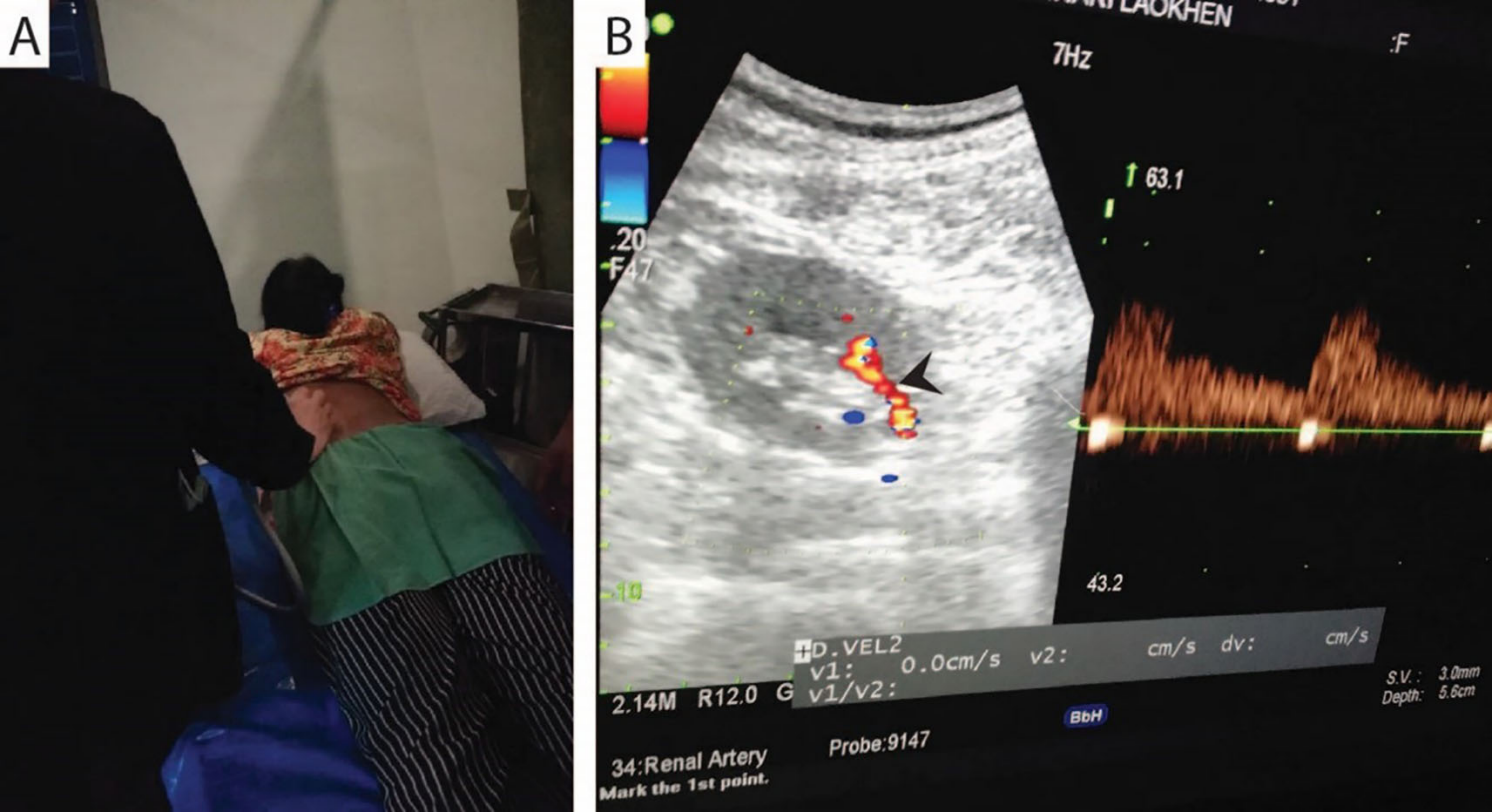
Examination of renal blood flow by a radiologist using Doppler ultrasound (A). Color flow ultrasound image demonstrates a representative left renal artery (B, arrowhead).

The RBF parameters, including peak systolic velocity (PSV), end-diastolic velocity (EDV), resistive index (RI), and volumetric arterial blood flow (VF), were measured in a prone lying position with a linear 4.4-13 MHz probe of Doppler ultrasound (ProSound F75 continuous-wave ultrasound, Hitachi-Aloka). The ultrasound probe was placed between the 12 ^th^ thoracic vertebra and the 2 ^nd^ lumbar vertebra, 5 cm lateral to the spine, to measure blood flow of the left renal artery. The RI was calculated from PSV-EDV/PSV. ^12^ The VF is equal to cross-sectional area (A) x time-averaged velocity (TAV). As previously described, the A and TAV can be calculated as π x radius. ^2^^,^^13^ These parameters were measured twice, and the mean of each parameter was recorded. RBF was measured immediately after the measurement of HRV parameters by the radiologists ( Figure 4).

### Sample size calculation

With an effect size of 1.2, ^14^ α-error level (two-sided)=0.05, and 1-β error level=0.95, the G*power algorithm was used to determine the sample size for each group, yielding n=12/group.

### Statistical analysis

The data were expressed as mean±standard error of the mean (SEM). Shapiro–Wilk test was used to verify normal distribution. Paired t-test was used to compare the outcome variables between before and after experiments within a group. An independent t-test was used to compare outcome variables between groups. Statistical significance was set at the P<0.05.

## Results

The remaining twenty-six volunteers were divided into two groups, the TFM group (13 persons; female=11, male=2), with an average age of 68.15±1.52 years old. They received a 15-minute Thai foot massage using moderate thumb press along three lines on both feet in a supine lying position ( Figure 2). The control group (13 persons; female=10, male=3) had an average age of 65.00±1.10 years old. They received a 15-minute bed rest in the same position. The volunteers were advised to refrain from eating, drinking alcohol, smoking, and consuming caffeine for at least 2 hours before participating in this study. Their basal renal function (urinary creatinine excretion rate) and predicted muscle mass were not significantly different.

Within-group comparison of the means between before and immediately after experiments demonstrated that VF was significantly increased only in the TFM group (p=0.012) but not in the control group. PSV, EDV, and RI were not changed in both groups. No side effects were observed.

Data values are expressed as mean±SEM. Thai foot massage (TFM) values are compared to control values with independent t-tests.

Data values are expressed as mean±SEM. Pre-intervention values are compared to post-intervention values with paired t-tests. RBF parameters include volumetric arterial blood flow (VF), peak systolic velocity (PSV), end-diastolic velocity (EDV), and resistive index (RI).

SDNN, RMSSD, and HF were significantly increased in the TFM group (p=0.000, p=0.020, p=0.019, respectively). RMSSD was significantly increased in the C group (p=0.032). However, LF and LF/HF ratio were not changed in the TFM group, and SDNN, HF, LF, and LF/HF ratio were not altered in the C group ( Table 1 and Table 2). The between-group comparison showed no difference between the groups in all parameters.

**Table 1.** Basic characteristics of participants.

Characteristics	Control group (n=13)	TFM group (n=13)	t	P-value
Male/Female	3/10	2/11		
Age (years)	65.00±1.10	68.15±1.52	-1.680	0.106
Weight (kg)	56.92±3.02	54.30±1.84	0.740	0.466
Height (cm)	153.69±2.49	152.00±1.49	0.583	0.565
BMI (kg/m ^2^)	24.27±1.44	23.55±0.85	0.432	0.669
Systolic blood pressure (mmHg)	140.69±4.23	143.08±4.30	-0.396	0.696
Diastolic blood pressure (mmHg)	76.08±3.19	72.31±2.93	0.870	0.393
Urine volume (ml)	964.73±81.52	1000.26±83.44	-0.304	0.763
Urinary creatinine excretion rate (mmol/24 h)	5.76±0.67	5.49±0.36	0.366	0.718
Predicted muscle mass (kg)	16.42±1.31	15.84±0.76	0.376	0.711

**Table 2.** Pre-intervention and post-intervention renal blood flow (RBF) parameters in control and TFM groups.

RBF parameter	Control group (n=13)		t	P-value	TFM group (n=13)		t	P-value
	Pre-intervention	Post-intervention			Pre-intervention	Post-intervention		
VF (mL/min)	527.00±114.33	479.39±104.47	1.07	0.85	412.27±46.07	491.12±53.62	-1.90	0.04
PSV (cm/s)	55.11±5.03	50.27±4.34	1.76	0.95	50.15±3.90	52.15±3.80	-0.84	0.21
EDV (cm/s)	15.41±1.83	16.24±1.81	-0.61	0.28	13.77±1.71	14.24±0.95	-0.31	0.38
RI	0.73±0.02	0.72±0.02	0.41	0.66	0.73±0.02	0.74±0.03	-0.36	0.36

HRV parameters include stress index, standard deviation of the normal-to-normal intervals (SDNN), root mean square of successive differences (RMSSD), high frequency (HF), low frequency (LF), and low frequency per high frequency (LF/HF) ratio ( Table 3).

**Table 3.** Pre-intervention and post-intervention heart rate variability (HRV) parameters in control and TFM groups.

HRV parameters	Control group (n=13)		t	P-value	TFM group (n=13)		t	P-value
	Pre-intervention	Post-intervention			Pre-intervention	Post-intervention		
Stress index	59.81±2.50	57.58±2.23	1.35	0.10	66.85±2.87	61.65±3.42	2.44	0.02
SDNN (ms)	33.59±4.05	36.90±3.83	-1.32	0.11	25.93±2.99	30.52±3.07	-2.92	0.00
RMSSD (ms)	29.25±4.12	32.25±3.80	-2.90	0.00	22.09±2.80	26.09±3.36	-1.87	0.04
HF (ms ^2^)	5.09±0.22	5.21±0.18	-1.31	0.11	4.62±0.25	4.88±0.26	-1.64	0.06
LF (ms ^2^)	5.51±0.22	5.62±0.17	-0.63	0.27	4.86±0.26	5.27±0.28	-2.37	0.02
LF/HF ratio	1.08±0.04	1.08±0.02	-0.10	0.54	1.07±0.05	1.10±0.06	-0.64	0.73

## Discussion

This study demonstrated that TFM increased renal blood flow and reduced psychological stress through parasympathetic nervous system activation in older adults. Our statement is based on the following findings. First, the TFM increased the VF by approximately 20%. Secondly, the TFM significantly reduced the stress index. Lastly, the TFM increased the SDNN and RMSSD, the proxies of the parasympathetic activity.

Doppler ultrasonography is a widely used non-invasive technique for examining the morphology and function of the intraparenchymal vascularity of the kidney. ^15^ The VF can be estimated using the Doppler approach, which involves multiplying the flow velocity by the cross-sectional lumen diameter at that exact moment in time. ^16^ Using the magnetic resonance imaging (MRI) method, the renal artery VF reportedly ranged from 200 to 1200 mL/min. ^17^ The VF values in the present study (400-500 mL/min) are within the normal limits. Alternatively, the blood flow velocity can also be measured as an index of vascular function. A previous study by Iwamoto and colleagues demonstrated that a two-minute friction massage around the halfway point of the gastrocnemius muscle’s medial and lateral heads increased the popliteal venous blood flow velocity. ^18^ Our present finding of the increment of approximately 20% of the VF after the TFM aligns with our previous report on the stimulatory effect of active ankle movements on the VF. ^19^ The mechanism of the massage-induced VF increase is unclear, but the previous study showed that passive muscle movement could increase cerebral blood oxygenation in older persons. ^20^ Therefore, the TFM probably improves the VF of the renal artery in the same way. Previously, the 8-min reflexology-based foot massage was reported to reduce the RI of the renal artery. However, the RI and other RBF parameters (PSV and EDV) were not altered by the TFM in the present study.

We found that the PSV and EDV in the renal artery are approximately 50-55 and 13-16 cm/s, respectively. These values are lower than the typical ranges of previously reported PSV (60-100 cm/s) and EDV (20-50 cm/s). ^21^^,^^22^ Previous studies showed that the PSV and EDV in older adults were approximately 60-70 and 19-21 cm/s. ^23^ Consequently, our lower PSV and EDV findings resulted in elevated RI (> 0.7). ^24^ The exalted RI might be explained by the averaged participant’s high blood pressure in the present study, as shown in over two-thirds of hypertensive patients without renal artery stenosis. ^25^ Notably, those with RI ≥ 0.70 had a lower estimated glomerular filtration rate (eGFR) than those with RI < 0.70. ^25^

As mentioned, the RBF is closely related to psychological stress. The psychological stress leads to renal vasoconstriction, discernibly due to increased sympathetic nervous activity in the kidneys. ^26^ Moreover, a higher degree of psychological stress has been linked with a greater likelihood of an accelerated eGFR decline. ^27^ Based on our present finding of the TFM-reducing stress index, it is tempting to further inspect the relationship between the RBF, psychological stress, and autonomic nerve activities in a chronic kidney disease subpopulation group.

Our findings of TFM-induced SDNN and RMSSD indicate an increase in parasympathetic activity. These observations accord with the previous report on the parasympathetic nervous system stimulation by foot reflexology, which also causes the release of endogenous substances. According to this idea, localized enzymatic reactions in receptive fields and skin-to-skin contact raise localized skin temperatures, enhancing physical function and blood flow. ^28^^,^^29^ It has also been shown that foot massage increases vagal activity in older adults with heart disease, confirming that it is beneficial and safe even in vulnerable individuals. ^30^ Of note, we also found that the TFM increased the LF component of the HRV, implying sympathetic activation.

However, the summed effect of the parasympathetic activity is probably more predominant, as shown by the reduced stress index. There are a few limitations in the present study. First, the majority of the volunteers were female. Generally, the RBF in females is 20% lower than in males. ^1^ A larger sample size in a further study might be stratified into two groups to observe the gender difference. Secondly, molecular mechanisms of the TFM were not directly measured. In the other study, biochemical markers from blood samples, such as vasodilators, can be determined. ^31^ Our analysis is confined to older adults, so generalization should be performed carefully.

In summary, by activating parasympathetic activity, the TFM may raise RBF and reduce psychological stress. As a result, this intervention may reduce stress in the senior population and enhance RBF. It is necessary to conduct additional research on a broader population.

## Data Availability

OSF: Repositories for a project: Immediate effects of Thai foot massage on renal blood flow and heart rate variability in older adults. https://doi.org/10.17605/OSF.IO/YRF3J Data are available under the terms of the Creative Commons Zero “No rights reserved” data waiver (CC0 1.0 Public domain dedication). This project includes the following underlying data.
- Data-1.xlsx OSF: Repositories for a project: Immediate effects of Thai foot massage on renal blood flow and heart rate variability in older adults. https://doi.org/10.17605/OSF.IO/YRF3J Data are available under the terms of the Creative Commons Zero “No rights reserved” data waiver (CC0 1.0 Public domain dedication). This project includes the following extended data.
- A copy of the original trial protocol.pdf - CONSORT flow diagram.tiff - CONSORT-2010-Checklist-revised-1.pdf OSF: CONSORT-2010-Checklist-revised-1.pdf in Repositories for a project: Immediate effects of Thai foot massage on renal blood flow and heart rate variability in older adults. https://osf.io/yrf3j/ Data are available under the terms of the Creative Commons Zero “No rights reserved” data waiver (CC0 1.0 Public domain dedication). CONSORT flow diagram.tiff
- CONSORT-2010-Checklist-revised-1.pdf
