# Supplementary material for: Immediate effects of Thai foot massage on renal blood flow, psychological stress, and heart rate variability in community-dwelling older adults: a randomized controlled trial
Source: F1000Res. 2024 Jul 26;13:838. [Version 1] doi: 10.12688/f1000research.148453.1 (PMC11576562; doi:10.12688/f1000research.148453.1)
Supplement: Peer review history

# Reviewer report: Reviewer response for version 1

**DOI:** 10.5256/f1000research.162762.r338484

**Published:** 2024-11-19

**Authors:** Enggista Hendriko Delano, Sabda Hussain As Shafi, Wahyu Aji Nugroho

**Affiliations:** 1 Universitas Negeri Yogyakarta, Yogyakarta, Indonesia; 2 Sport Science, State University of Yogyakarta (Ringgold ID: 148002), Yogyakarta, Special Region of Yogyakarta, Indonesia

Review:

1. Study this is very good , Purposeful For determine the effects of Thai foot massage on renal blood flow, psychological stress, and heart rate variability in community-dwelling older adults.
2. Background​ study it's very clear
3. Compliance between objective with method research used​  are already appropriate.
4. Election sample is already determined based on criteria and quantity sample used to calculate the sample size. Does the sample take into consideration variables such as type and sex?
5. Measuring instruments used​ need listed with clear along with mark validity and reliability.
6. The intervention section needs further clarification regarding the Thai massage treatment steps, including which body parts are involved, which muscles and points are affected, and the direction of the massage. If possible, images and tables can be added to provide a clearer understanding of the treatment steps.
7. The results section would be improved by adding a clear indication of the effectiveness based on the study's findings
8. Clarification is needed on why massage on the soles of the feet can provide effective therapy. This should be explained and supplemented with the relevant physiological mechanisms of the body.

Is the work clearly and accurately presented and does it cite the current literature?

Yes

If applicable, is the statistical analysis and its interpretation appropriate?

Yes

Are all the source data underlying the results available to ensure full reproducibility?

Yes

Is the study design appropriate and is the work technically sound?

Yes

Are the conclusions drawn adequately supported by the results?

Yes

Are sufficient details of methods and analysis provided to allow replication by others?

Yes

Reviewer Expertise:

Sport Therapy, Sport Massage, Complementary therapy for sports injuries.

We confirm that we have read this submission and believe that we have an appropriate level of expertise to confirm that it is of an acceptable scientific standard.

# Reviewer report: Reviewer response for version 1

**DOI:** 10.5256/f1000research.162762.r308010

**Published:** 2024-08-08

**Authors:** Orachorn Boonla

**Affiliations:** 1 Faculty of Allied Health Sciences,Exercise and Nutrition Innovation and Sciences Research Unit, Burapha University, Mueang Chonburi District, Chon Buri, Thailand

This study demonstrated that Thai foot massage increases renal blood flow and reduces psychological stress through parasympathetic nervous activation in older adults.

 This study provided adequate background and information to help you understand the research. The author provided adequate findings that correlate with the results and the conclusions align with the findings obtained from the study.

 However, the author should provide the full name of the abbreviation at the end of each figure and table such as VF, PSV, EDV, and RI. It is necessary for the author to review the results in Table 3. The results show a significant difference in HRV parameter (LF) in TFM group. The manuscript should be indexed with a few minor changes.

Is the work clearly and accurately presented and does it cite the current literature?

Yes

If applicable, is the statistical analysis and its interpretation appropriate?

Yes

Are all the source data underlying the results available to ensure full reproducibility?

Yes

Is the study design appropriate and is the work technically sound?

Yes

Are the conclusions drawn adequately supported by the results?

Yes

Are sufficient details of methods and analysis provided to allow replication by others?

Yes

Reviewer Expertise:

Cardiovascular physiology, Oxidative stress, Exercise physiology, Geriatic physical therapy

I confirm that I have read this submission and believe that I have an appropriate level of expertise to confirm that it is of an acceptable scientific standard.
